# Prophylactic Addition of Glucose Suppresses Cyanobacterial Abundance in Lake Water

**DOI:** 10.3390/life12030385

**Published:** 2022-03-07

**Authors:** Stephen Vesper, Nathan Sienkiewicz, Ian Struewing, David Linz, Jingrang Lu

**Affiliations:** United States Environmental Protection Agency, 26 W. Martin Luther King Drive, Cincinnati, OH 45268, USA; sienkiewicz.nathan@epa.gov (N.S.); struewing.ian@epa.gov (I.S.); linz.david@epa.gov (D.L.); lu.jingrang@epa.gov (J.L.)

**Keywords:** glucose, cyanobacteria, proteobacteria, *Microcystis aeruginosa*, microcystin, prophylactic

## Abstract

To mitigate harmful cyanobacterial blooms (HCBs), toxic algicides have been used, but alternative methods of HCB prevention are needed. Our goal was to test the prophylactic addition of glucose to inhibit HCB development, using *Microcystis* and the toxin microcystin as the HCB model. Water samples were collected weekly, from 4 June to 2 July, from Harsha Lake in southwestern Ohio during the 2021 algal bloom season. From each weekly sample, a 25 mL aliquot was frozen for a 16S rRNA gene sequencing analysis. Then, 200 mL of Harsha Lake water was added to each of the three culture flasks, and glucose was added to create concentrations of 0 mM (control), 1.39 mM, or 13.9 mM glucose, respectively. The microcystin concentration in each flask was measured after 1 and 2 weeks of incubation. The results showed an 80 to 90% reduction in microcystin concentrations in glucose-treated water compared to the control. At the end of the second week of incubation, a 25 mL sample was also obtained from each of the culture flasks for molecular analysis, including a 16S rRNA gene sequencing and qPCR-based quantification of *Microcystis* target genes. Based on these analyses, the glucose-treated water contained significantly lower *Microcystis* and microcystin producing gene (*mcy*) copy numbers than the control. The 16S rRNA sequencing analysis also revealed that Cyanobacteria and Proteobacteria were initially the most abundant bacterial phyla in the Harsha Lake water, but as the summer progressed, Cyanobacteria became the dominant phyla. However, in the glucose-treated water, the Cyanobacteria decreased and the Proteobacteria increased in weekly abundance compared to the control. This glucose-induced proteobacterial increase in abundance was driven primarily by increases in two distinct families of Proteobacteria: *Devosiaceae* and *Rhizobiaceae*. In conclusion, the prophylactic addition of glucose to Harsha Lake water samples reduced Cyanobacteria’s relative abundance, *Microcystis* numbers and microcystin concentrations and increased the relative abundance of Proteobacteria compared to the control.

## 1. Introduction

Harmful cyanobacterial blooms (HCBs), and the toxins they produce, are a threat to the safety of drinking and recreational water and the aquatic ecosystem [1,2]. To prevent HCBs, it is necessary to limit the introduction of high concentrations of fixed nitrogen compounds and phosphates [3]. Until that happens, agents or methods are needed that can limit the production of cyanotoxins. 

Most HCB control measures involve the addition of an algicide once the bloom has developed. Many HCB control agents can reduce cyanotoxin concentrations but these algicides are dangerous to handle and toxic. Algicides usually contain hydrogen peroxide, sometimes combined with an acid such as peroxyacetic acid, or the algicide is a formulation of copper (Cu) [4,5,6,7]. Hydrogen peroxide is toxic at high concentrations and is a strong oxidizer, which makes it dangerous to handle [8]. Copper is a toxic metal that can bioaccumulate in freshwater ecosystems [9]. Other control methods are in development.

Biological control measures, such as plant-derived allelochemicals [10] or other biologically derived molecules [11], have been tested. Algal predation by bacteria [12] and algicidal bacteria [13] have also been evaluated. Even algicidal and toxin-degrading fungi have been tested [14,15]. Physical measures have also been evaluated, including the photodegradation of algal toxins [16]. However, these newer control strategies do not have a broad efficacy [17]. 

The goal of this study was to test the prophylactic addition of glucose in preventing/controlling HCB development as well as to understand its impact on microbial community dynamics following treatment. Our hypothesis is that the excess fixed nitrogen and phosphates would be utilized by non-toxin-producing microorganisms if they had an adequate carbon source. By introducing a carbon source before the bloom develops, these other microorganisms might be able to out-compete the algae and suppress their growth. 

*Microcystis* is the cyanobacterium most often identified in freshwater blooms that becomes harmful because they produce a class of cyanotoxins called microcystins [1,3,18,19,20]. Therefore, *Microcystis* and the cyanotoxin, microcystin, were used as a model in this study. 

## 2. Materials and Methods

### 2.1. Study Site

The William H. Harsha Lake (hereafter, Harsha Lake) is an engineered reservoir (latitude: 39.0132285, longitude: −84.1148988) that was developed in 1978. Harsha Lake is located on the East Fork of the Little Miami River in Clermont County, Ohio, about 40 km east of Cincinnati. Harsha Lake has an average depth of 13.1 m, with a maximum depth of 34 m. Harsha Lake covers an area of 8.7 km^2^ and drains from a watershed of 890 km^2^ with 64% of land use for agriculture and 26% comprised of forest cover [21]. The development of algal blooms in Harsha Lake has been studied by our team since 2015 [21,22,23]. Water collected from Harsha Lake during the 2021 bloom season was used for these studies.

### 2.2. Collection of Harsha Lake Samples

Harsha Lake water samples were collected weekly, from 4 June to 9 July 2021, using plastic water jugs, which were pre-rinsed using 5% hydrochloric acid and deionized water, in order to scoop up water from the surface (~0.5 m depth) at 3 locations, as previously described [21]. The samples from each location were combined each week to make a single composite sample for testing in that particular week. This composite was defined as the “raw water” sample. A 25 mL aliquot of the raw water was removed each week and frozen at −20 °C for molecular analysis. Then, 200 mL of the raw water sample was added to each of the 3 cell culture flasks (661–175, CELLSTAR, Frickenhausen, Germany). Then, D-(+)-glucose (G7528, Sigma Ultra, Sigma-Aldrich, St. Louis, MO, USA) was added to make a final glucose concentration in each flask of either 0 mM, 1.39 mM or 13.9 mM glucose per liter. 

### 2.3. Incubation of Treated Water Samples 

Flasks were incubated in an environmental chamber (Percival/166LLVL) with the following growth conditions: the light intensity was 44.02 µmol photons/m^2^/s (measured using a LICOR LI-1500 (LI COR Biosciences, Lincoln, NE, USA) with a 16/8 h light/dark cycle at a constant temperature of 25 °C and ambient air exchange. After 1 week and 2 weeks of incubation, 3 replicate 5 mL samples were collected and placed into conical bottom glass tubes (PYREX 15 mL centrifuge tube, Corning Inc., Corning, NY, USA). Samples were frozen at −20 °C until an analysis of microcystin concentrations. At the end of 2 weeks of incubation, a 25 mL sample was removed from each of the 3 treatment flasks and frozen at −20 °C for later analysis. 

### 2.4. Analysis of Microcystin Using Enzyme-Linked Immunosorbent Assay (ELISA)

Measurements of microcystin (MC) were measured in raw and filtered water using the MC-ADDA Enzyme-Linked Immunosorbent Assay (ELISA) kit (PN 520011, Abraxis, Warminster, PA, USA), following the manufacturer’s instructions. The 5 mL subsamples were collected in glass tubes (PYREX 15 mL centrifuge tube, Corning, NY, USA) and frozen at −20 °C until processing and analysis. Total microcystin concentrations were measured by subjecting each sample to 3 freeze–thaw cycles. Assays were performed manually and analyzed using a Biolog Microstation plate reader (Biolog, Hayward, CA, USA).

### 2.5. DNA Extraction and High-Throughput Sequencing

The samples frozen for molecular analysis were filtered through a Durapore polyvinylidene fluoride (PVDF) filter (0.45 μm, MilliPore, Foster City, CA, USA). Each filter was then inserted into 1.5-mL microtube that contained 1 mL TRIzol reagent (Thermo-Fisher, Waltham, MA, USA) and stored at −80 °C until analyzed. 

Filters were disrupted, and cells lysed using a Mini-Beadbeater-16 (BioSpec Products, Inc., Bartlesville, OK, USA) twice for 30 s and then centrifuged at 10,000× *g* for 3 min. The supernatant was then transferred to a new sterile tube, and RNA and DNA were extracted and purified from the TRIzol reagent, following the manufacturer’s instructions. The extracted RNA and DNA was eluted in 200 µL RNase-free water (Sigma-Aldrich, St. Louis, MO, USA).

The bacterial 16S gene primers used for 16S rRNA gene sequencing were designed by using the V3 and V4 regions [24] and an adapter sequence was added, as described previously [25]. Library preparation and sequencing were performed, as described [25] with the following modifications. The first round PCR was performed with 17 µL Accuprime pfx supermix (Thermo-Fisher, Waltham, MA, USA), 0.5 µL of each primer, and 2 µL of DNA. After gel confirmation of amplification products, PCR products were cleaned with 14 µL of AMPure XP beads (Beckman Coulter, Brea, CA, USA) to 17 µL PCR product and eluted in 40 µL of 10 mM Tris pH 8.5. PCR products were normalized to 20 ng/µL and Index PCR performed using Accuprime pfx supermix, cleaned using 19 µL AMPure XP to 17 µL of PCR product, and eluted with 27 µL of 10 mM Tris pH 8.5. Samples were normalized to a concentration of 4 nM, and 5 µL of each were combined to make the final library. The library was sequenced using a 600-cycle V3 MiSeq sequencing kit (# MS-102-3003, Illumina, San Diego, CA, USA) according to the manufacturer’s protocol and using 2 × 300 paired-end sequencing. 

The assay used to quantify total *Microcystis* cells, 16SMic, was published previously [26]. The McyEmic is a qPCR assay that specifically quantifies *Microcystis* cells that produce the microcystin toxin. Because there is only one copy in each toxic *Microcystis* cell*,* its abundance is also the quantity of toxic *Microcystis* cells. The primers used to quantify McyEmic were as follows: McyEmicF-GTTATGTTTGCCGGCTCCTA and McyEmicR-GTGCCTAGACTTAAGGGTTGAG. 

The qPCR reaction mixtures (20 μL) contained 10 μL 2× qPCR SYBR Green Master Mix (Life Technologies, Carlsbad, CA, USA), 0.2 μM primers (final concentration) and 2 μL of template DNA. Initial DNA treatment consisted of 50 °C for 2 min with UNG (Uracil-N-Glycosylase) to prevent carryover contamination, followed by 95 °C for 10 min for DNA denaturing. The quantification cycling protocol, using a QuantStudio™ 6 Flex system (Life Technologies), was 40 cycles at 95 °C for 15 s followed by one cycle for 1 min at either 60 °C for the McyEmic assay or at 64 °C for the 16sMic assay, then followed by the default melt curve analysis. The quantification cycling protocol, using a QuantStudio™ 6 Flex system (Life Technologies), was 40 cycles at 95 °C for 15 s followed by one cycle for 1 min at either 60 °C for the McyEmic assay or at 64 °C for the 16sMic assay, then followed by the default melt curve analysis. DNA copies were quantified relative to a standard curve using linearized plasmids constructed from PCR products from *M. aeruginosa* genomic DNA cloned into a PCR 4-TOPO vector (Invitrogen, Waltham, MA, USA). Each sample was run in duplicate, and the amplicons were pooled. Each qPCR plate contained a triplicate six-point standard curve with values ranging from 10^1^ to 10^6^ copies. 

### 2.6. Amplicon Processing

Pooled amplicons were sequenced using an Illumina MiSeq and 300 bp paired-end chemistry (Illumina). Raw demultiplexed reads, with adapters removed, were then processed using the software suite QIIME 2 2021.4.0 [27]. Raw sequence data were quality filtered and denoised with DADA2 [28] (via q2-dada2). Taxonomy was assigned to amplicon sequence variants (ASVs) using the q2-feature-classifier [29] and classify-sklearn naïve Bayes taxonomy classifier against the Silva 138 99% operational taxonomic units (OTUs) reference sequences [30,31,32]. Qiime2 artifacts were then moved to R using the qiime2R package for further analysis [33]. All data were deposited in the National Center for Biotechnology Information (NCBI) sequence read archive with the accession number: PRJNA786865.

### 2.7. Data Analysis

Analysis of the final sequence dataset was performed in R v4.1.2 [33] (R Core Team, 2021) using the packages phyloseq [34], vegan [35], and ggplot2 [36]. Samples were initially pruned of non-bacterial or unidentified taxa, including those amplicon sequence variants (ASVs) classified as belonging to the order “Chloroplasts”. For certain components of our analysis, low abundance taxa (below 1% relative abundance) were removed from the dataset. Bray–Curtis between-sample distances were computed. Distance matrices were then used to cluster samples using non-metric multidimensional scaling (NMDS). Analysis of similarities (ANOSIMs) on the distance matrices were used to test for statistically significant differences in the microbiota composition and diversity between sample groups [37]. Differences were compared between the control and 13.9 mM treatment at the 95% confidence level.

## 3. Results

### 3.1. Microcystin Concentrations after Glucose Treatments

**Figure 1:**
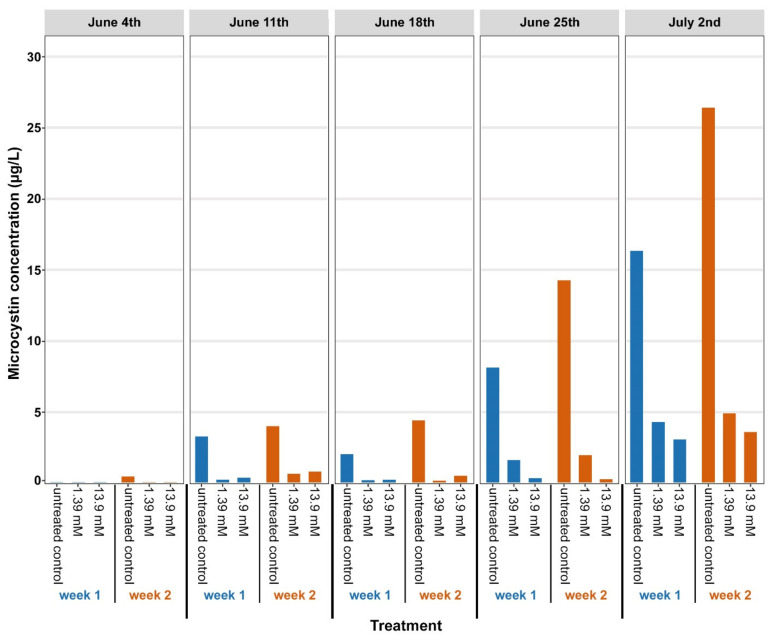
Total microcystin concentrations in Harsha Lake water samples after glucose treatment. Harsha Lake water samples were collected weekly from 4 June to 2 July and left untreated (controls), or they were treated with 1.39 mM or 13.9 mM glucose. After 1 week (blue color) or 2 weeks (orange color) of incubation, microcystin concentrations were quantified by ELISA. Glucose treatment consistently reduced microcystin concentrations.

The concentration of microcystin in the culture flasks after 1 week or 2 weeks of incubation are shown in Figure 1 for each collection week of the study. The culture flasks with a final glucose concentration of either 1.39 mM or 13.9 mM glucose contained significantly (Student *t*-test, *p* < 0.05) lower concentrations of microcystin each week compared to the control. 

**Figure 2:**
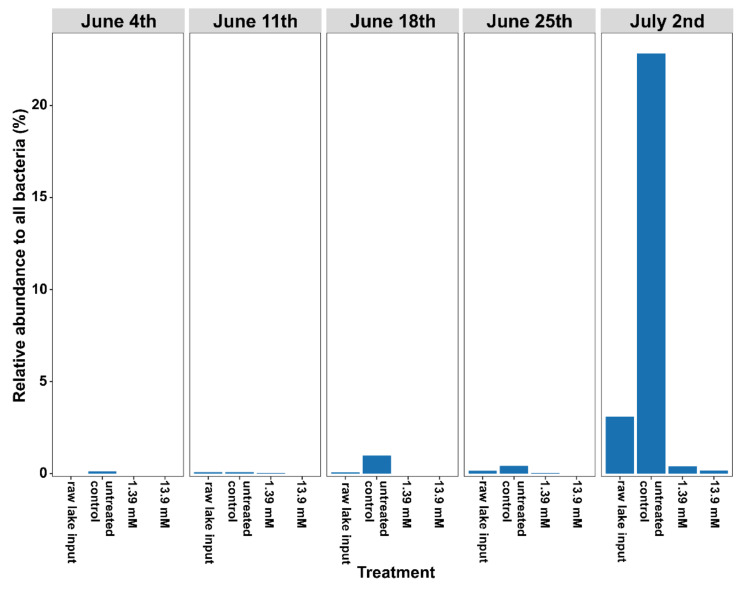
The relative abundance of taxa belonging to the genus *Microcystis* in the weekly Harsha Lake samples, after glucose treatments. Harsha Lake water samples were collected weekly from 4 June to 2 July and sequenced for 16S rRNA gene analysis (raw lake input). Raw lake samples were then left untreated (untreated controls) or treated with 1.39 mM or 13.9 mM glucose for each sample date. After 2 weeks of incubation, untreated controls and glucose-treated samples were also sequenced. Relative abundance of *Microcystis* sp., as determined by 16S sequencing, was reduced after glucose treatment despite increasing prevalence at later lake sample points and rapid growth of these taxa in untreated cultures.

These differences in microcystin concentrations were further supported by quantifying *Microcystis* toxin gene copy number by qPCR. This approach showed decreases in toxin gene abundance after glucose treatment parallel to the toxin quantification (Table 1). In addition, we also confirmed these findings via the metagenomic-based quantification of the *Microcystis* genus (Figure 2). By the week of 9 July, the Harsha Lake algal bloom was fully developed based on field observations. However, within 3 days of the placement of the 9 July water sample flasks in the incubator, it was evident from their appearance that the cultures’ survival at these high densities could not be sustained in the laboratory, and the experiment was terminated.

### 3.2. Changes in Microbial Community Structure Based on 16S rRNA Gene Analysis

**Figure 3:**
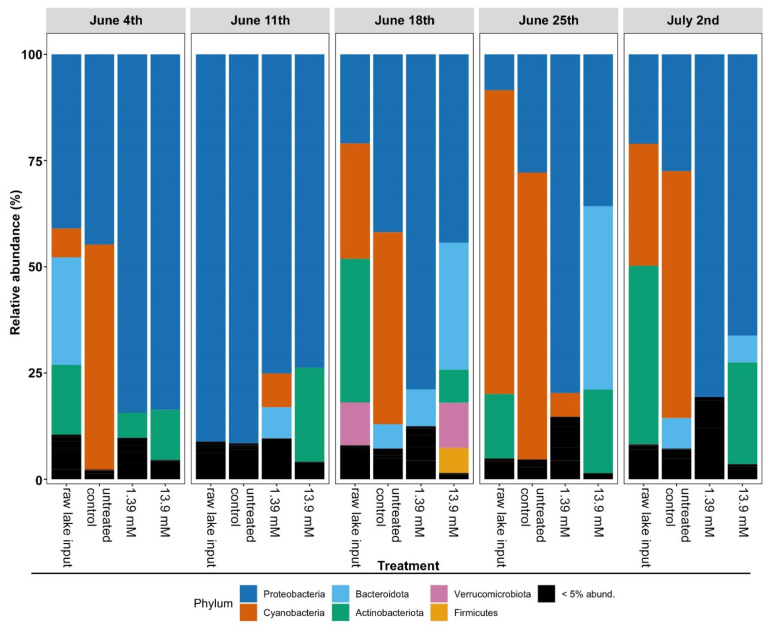
The relative abundance of bacterial phyla in the weekly Harsha Lake samples, after 2 weeks of incubation. Harsha Lake water samples were collected weekly from 4 June to 2 July and sequenced for 16S rRNA gene analysis (raw lake input). Raw lake samples were then left untreated (untreated controls) or treated with 1.39 mM or 13.9 mM glucose for each sample date. After 2 weeks of incubation, untreated controls and glucose-treated samples were also sequenced. Relative abundance of bacterial phyla was determined for each sample using 16S sequencing. Any phyla occurring below 5% relative abundance were compiled as a single group (black bars).

To comprehensively profile the microbial communities that occur in the Harsha Lake samples, 16S rRNA gene sequencing was performed both before and after glucose treatment. Across all samples, only six phyla occurred above 5% relative abundance in any given sample (Figure 3). The phyla, Cyanobacteria and Proteobacteria, were the most prevalent across all samples (Figure 3). As the summer progressed, Cyanobacteria prevalence increased and became the dominant phyla in the Harsha Lake water and untreated samples (Figure 3). However, the Proteobacteria consistently increased in the glucose-treated water samples compared to the controls (Figure 3). Therefore, the changes in the relative abundance of Cyanobacteria and Proteobacteria were the focus of further analyses.

**Figure 4:**
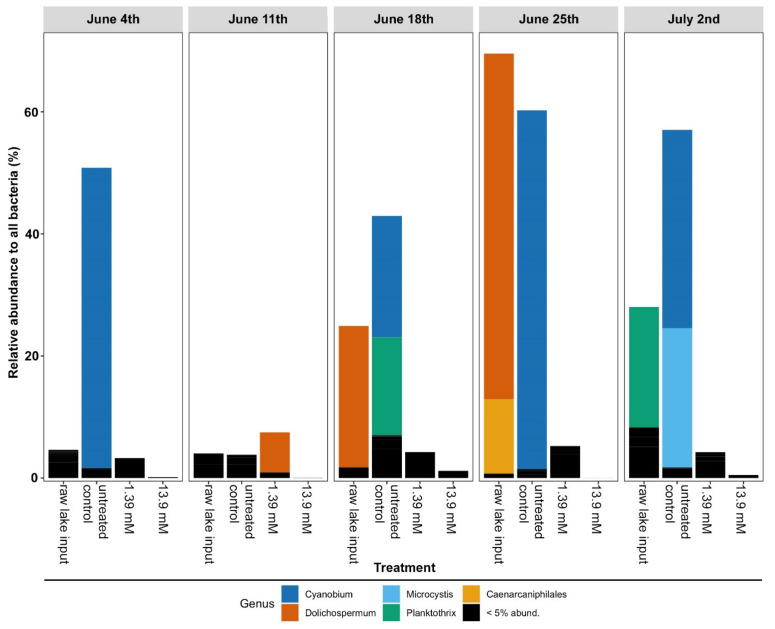
The relative abundance of genera within the phylum Cyanobacteria in the weekly Harsha Lake samples after 2 weeks of incubation. Harsha Lake water samples were collected weekly from 4 June to 2 July and sequenced for 16S rRNA gene analysis (raw lake input). Raw lake samples were then left untreated (untreated controls) or treated with 1.39 mM or 13.9 mM glucose for each sample date. After 2 weeks of incubation, untreated controls and glucose-treated samples were also sequenced. Relative abundance of cyanobacterial genera was determined for each sample using 16S sequencing. Any phyla occurring below 5% relative abundance were compiled as a single group (black bars).

A closer examination of Cyanobacterial abundance showed that there were 32 Cyanobacterial genera that occurred in these lake water samples; however, only a few genera occurred above a 5% relative abundance in any given sample (Figure 4). In the initial lake water sample and in the samples without glucose, three cyanobacterial genera occurred at the highest level: *Cyanobium*, *Dolichospermum,* and *Planktothrix* (Figure 4). In the 2 July weekly sample, *Microcystis* became one of the dominant genera. Importantly, across all 5 weeks of samples, the addition of glucose at 1.39 or 13.9 mM consistently resulted in a reduction in the abundance of all Cyanobacteria (Figure 4), along with the concomitant increase in the abundance of the Proteobacteria mentioned above (Figure 3). Therefore, the families of the Proteobacteria that underly these observed glucose-induced fluctuations were further investigated by examining sample differences, 13.9 mM vs. control flask, via ordination using non-metric multidimensional scaling (NMDS). 

**Figure 5:**
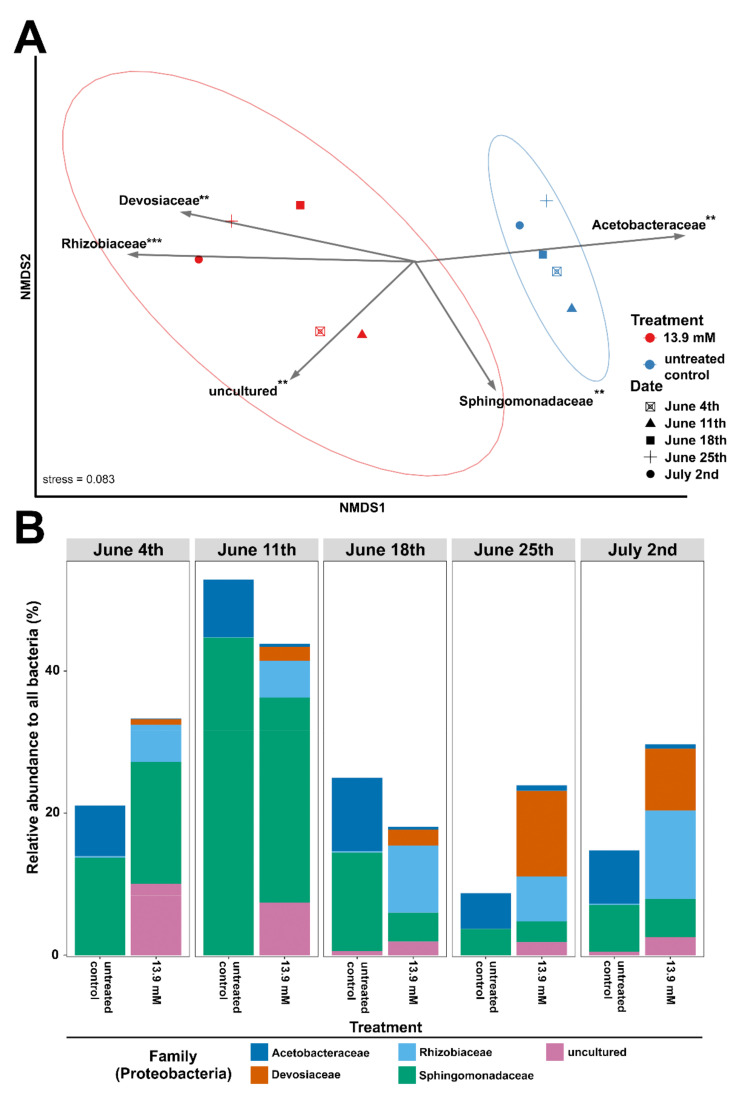
The changes in Proteobacterial taxa in the weekly Harsha Lake samples after 2 weeks of incubation. Harsha Lake water samples were collected weekly from 4 June to 2 July and then left untreated (untreated controls) or treated with 13.9 mM glucose for each sample date. After 2 weeks of incubation, untreated controls and glucose-treated samples were sequenced for 16S rRNA gene analysis. (**A**) Non-metric multidimensional scaling (NMDS) plot of Bray–Curtis distances for untreated controls (blue) and 13.9 mM glucose-treated samples (red) at each sample date (shapes) using the relative abundance of only Proteobacteria conglomerated at the family level. Proteobacterial family abundance was fitted as vectors to the NMDS using envfit. Only those with significant associations to NMDS clustering are shown. Arrow direction indicates increasing abundance and arrow length represents degree of significance. The ellipses represent 95% confidence intervals for each treatment. ** = *p* < 0.01, *** = *p* < 0.001. (**B**) Relative abundance of Proteobacterial families significantly fitted to NMDS in both untreated controls and 13.9 mM glucose-treated samples at each sample date.

Proteobacterial families were significantly clustered in a manner that reflected the glucose treatment (Bray–Curtis, ANOSIM; R = 0.912, *p* < 0.01), with no significant impact observed by date (Figure 5A,B). We also used envfit to fit individual Proteobacterial family abundance to our ordination. With this approach, we detected five families—*Devosiaceae*, *Rhizobiaceae*, *Sphingomonadaceae*, *Acetobacteraceae*, and a group of “unculturable” families—that significantly fit the data (Figure 5A). Among these families, it was primarily *Devosiaceae* and *Rhizobiaceae* (and “unculturable” families) that increased in abundance in the 13.9 mM glucose treatment (Figure 5A,B). 

## 4. Discussion

In this laboratory study of Harsha Lake water samples from the summer of 2021, the prophylactic addition of glucose resulted in a reduction in the abundance of Cyanobacteria and an increase in the abundance of Proteobacteria. Additionally, the abundance of the genus *Microcystis* and the toxin microcystin were reduced by the prophylactic addition of glucose. These results support our hypothesis that, given an external carbon source, heterotrophic bacteria could out-compete the cyanobacteria. 

Heterotrophic bacteria, such as Proteobacteria, have a high affinity for low nutrient concentrations, whereas phytoplankton such as Cyanobacteria are favored by pulses of high nutrient concentrations [38]. It appears that the addition of glucose in our study promoted heterotrophic bacterial growth, likely because they have higher nutrient-uptake rates than the Cyanobacteria [38,39,40] but are dependent on an external carbon source in their competition. Lu et al. [22] showed that, in Harsha Lake, nitrogen and phosphorous decreased rapidly during cyanobacterial growth from June to July. It appears that the addition of glucose to the water inhibited cyanobacterial nutrient uptake and, therefore, inhibited their growth. Additionally, Proteobacteria, including *Pseudomonas, Methylosinus, Methylobacterium*, *Sphingomonas* and others, are shown to degrade microcystin [41,42,43]. 

It is reasonable to infer that the outgrowth of Proteobacteria was likely due to the readily available carbon. Whether the addition of a carbon source will also decrease the dominance of other cyanotoxin producers, heterocystous cyanobacteria, or nitrogen fixers remains to be demonstrated. 

Our previous studies showed that N_2_ fixation was associated with high phosphorus but low nitrogen, as well as a high relative abundance of active cyanobacterial N_2_-fixers [22]. However, P-scavenging was associated with decreasing phosphorus and increasing *Microcystis abundance*. A similar shift in bacterial communities was also observed in a eutrophic lake in Utah [44]. The interplay between nitrogen fixation and phosphorus utilization provided an insight into the bacterial community structure and the understanding of bloom development [45]. Therefore, understanding these dynamics and knowing when to add a carbon source, such as glucose, is important.

We also previously showed that the detection of microcystin and microcystin producing algae by using qPCR and RT-qPCR analyses allowed for the rapid identification of a coming bloom and, potentially, the development of an early warning system [46]. By monitoring these dynamics, the optimal time for the prophylactic addition of glucose could be determined. However, the addition of glucose could have undesired effects.

One concern regarding the addition of glucose was that Proteobacteria that replaced Cyanobacteria might create another risk, i.e., enhanced levels of pathogenic bacteria. However, the two families in Proteobacteria that were most consistently amplified by the glucose treatment, *Devosiaceae* and *Rhizobiaceae*, do not contain significant human pathogens. However, we cannot rule out the possibility that human pathogens or zoonotic pathogens were amplified by the glucose addition. Another concern was that the addition of glucose in large quantities to a body of water will exacerbate eutrophication. Finding the minimal effective dose of glucose may reduce this impact. Controlling the bloom at the earliest stage may mean less potential ecological risks. The next studies will examine varying glucose concentrations and alternative carbon sources. 

One limitation of this study is that it was a pilot, bench-scale study. Nevertheless, Harsha Lake water samples were used, and the incubation was only for two weeks in the laboratory, thus minimizing any potential artifacts created by using the laboratory system. Another limitation of our study is that not all cyanobacteria were identified and quantified and not all cyanotoxins were measured. Whether glucose will have the same results for other genera and toxins remains to be tested. 

We recognize that there are many unknowns regarding the practical application of this approach to harmful cyanobacterial control. First, it is not clear under what circumstances this control method would be permitted. Adding large quantities of a carbon source to water could have unintended effects and these possibilities would need to be understood first. Additionally, it appears that the application of this method would be expensive because of the carbon source. Alternative carbon sources would need to be explored to reduce costs. Much research remains to be carried out to see if there is any situation where this method would be practical.

## 5. Conclusions

The prophylactic addition of glucose to Harsha Lake water samples reduced Cyanobacteria abundance, *Microcystis* numbers and microcystin concentrations and increased the abundance of Proteobacteria compared to the control. 

## Figures and Tables

**Table 1:** Gene copy number for the microcystin toxin gene (gene target, McyEmic) in *Microcystis* cells and total copies of *Microcystis* 16s ribosomal gene (gene target, 16SMic) in water samples at the end of 2 weeks of incubation. (SD = standard deviation; ND = not determined).

		Glucose Concentrations		
		0 mM	1.39 mM	13.9 mM
Sample Week	Gene Target	Copy Number ±SD/mL	Copy Number ±SD/mL	Copy Number ±SD/mL
6/4	McyEmic	1.6 ± 0.4 × 10^3^	0	0
	16SMic	1.1 ± ND × 10^3^	0	0
6/11	McyEmic	2.4 ± 0.8 × 10^3^	0	0
	16SMic	0.5 ± 0.1 × 10^3^	42 ± 4	0
6/18	McyEmic	5.7 ± 2.0 × 10^3^	0	0
	16SMic	6.2 ± 0.4 × 10^3^	0	0
6/25	McyEmic	1.3 ± 0.4 × 10^3^	30 ± 16	0
	16SMic	3.0 ± 0.7 × 10^3^	89 ± 15	0
7/2	McyEmic	1.6 ± ND × 10^5^	2.9 ± 2.2 × 10^3^	5.9 ± 4.9 × 10^3^
	16SMic	3.6 ± ND × 10^5^	0.9 ± 0.1 × 10^3^	0.8 ± ND × 10^3^

## Data Availability

All data will be available at the NIH-PMC website. All sequence data were deposited in the NCBI sequence read archive with accession number: PRJNA786865.
